# Residency Applicants Prefer Online System for Scheduling Interviews

**DOI:** 10.5811/westjem.2015.1.24615

**Published:** 2015-03-06

**Authors:** Charlotte Wills, H. Gene Hern, Harrison Alter

**Affiliations:** Alameda County Medical Center, Department of Emergency Medicine, Oakland, California

## Abstract

**Introduction:**

Residency coordinators may be overwhelmed when scheduling residency interviews. Applicants often have to coordinate interviews with multiple programs at once, and relying on verbal or email confirmation may delay the process. Our objective was to determine applicant mean time to schedule and satisfaction using online scheduling.

**Methods:**

This pilot study is a retrospective analysis performed on a sample of applicants offered interviews at an urban county emergency medicine residency. Applicants were asked their estimated time to schedule with the online system compared to their average time using other methods. In addition, they were asked on a five-point anchored scale to rate their satisfaction.

**Results:**

Of 171 applicants, 121 completed the survey (70.8%). Applicants were scheduling an average of 13.3 interviews. Applicants reported scheduling interviews using the online system in mean of 46.2 minutes (median 10, range 1–1800) from the interview offer as compared with a mean of 320.2 minutes (median 60, range 3–2880) for other programs not using this system. This difference was statistically significant. In addition, applicants were more likely to rate their satisfaction using the online system as “satisfied” (83.5% vs 16.5%). Applicants were also more likely to state that they preferred scheduling their interviews using the online system rather than the way other programs scheduled interviews (74.2% vs 4.1%) and that the online system aided them coordinating travel arrangements (52.1% vs 4.1%).

**Conclusion:**

An online interview scheduling system is associated with higher satisfaction among applicants both in coordinating travel arrangements and in overall satisfaction.

## INTRODUCTION

Residency applicants have to coordinate interviews at different programs even though interview offers may not be extended concomitantly. Residency administration may also be overwhelmed with scheduling residency interviews. Additionally, applicants are often doing rotations during “business hours,” which might prevent them from calling or reaching program coordinators in a timely fashion. We estimated scheduling and rescheduling interviews took 40hrs of administrator time the first week and 10hrs each subsequent week for the next three weeks each year.

A commercial online scheduling system that was available at all hours of the day might make the interview scheduling process easier for applicants and be a significant resource-saving investment for programs. An ideal system would allow both initial scheduling and would also allow changing/rescheduling of interviews later. Such a system might improve the experience for both applicants and for program coordinators. A prior study of a university-based, custom single site scheduling system, showed a 77% preference for scheduling interviews online.^1^

Our study objective was to determine applicant mean time to schedule and satisfaction using a commercially available Internet scheduling program. A secondary objective was to calculate the return on investment. We implemented this online program for the 2010–2011 residency interview season. Our program was one of four emergency medicine (EM) programs who used the system that year, the first year it was offered. We paid a fee of $2 per applicant for the use of this system and have no financial interest in it (www.Interviewbroker.com).

## METHODS

This pilot study is a retrospective analysis of applicants at an urban county EM residency. They received an anonymous survey asking about their experiences with the online interview scheduling system as compared with other programs. The survey was sent after interview offers were granted but before any interview occurred. Applicants were asked to provide the estimated time to schedule with the online system compared to the average time to schedule using other methods. They were asked to rate their satisfaction on a five-point anchored scale.

Our survey was developed and then piloted with departmental faculty with residency leadership experience. The survey and revisions were piloted with residents within our own program and in accordance with survey design methodology to maximize validity and reliability.^2^

Data analysis followed the assumption that questions regarding subjects’ experiences with and without the software should be treated as non-independent observations, and that the modified Likert scale should not be treated as an interval variable. With these assumptions, we analyzed Likert distributions using the Wilcoxon signed rank sum test. We also dichotomized the scale to “Satisfied” and “Less than satisfied,” which we analyzed with McNemar’s test for non-independent observations. The study instrument and protocol were approved by the institutional review board at our institution.

## RESULTS

Of 171 applicants, 121 completed the survey (70.8%). Applicants were scheduling an average of 13.3 interviews. Based on their responses, applicants estimated scheduling interviews using the online system took a mean of 46.2 minutes (median 10, range 1–1800) from the interview offer as compared with a mean of 320.2 minutes (median 60, range 3–2880) for other programs not using this system. This difference was statistically significant. In addition, applicants were more likely to rate their satisfaction using the online system as “satisfied” – 101 (83.5%) or “somewhat satisfied” – 16 (13.2%), as compared to “neither satisfied or unsatisfied” – 1 (0.8%) or “somewhat unsatisfied” – 2 (1.7%) or “unsatisfied” – 1 (0.8%).

Among applicants who had to change interview dates, 35 of 40 (90%) were “satisfied” in their ability to change their date. Among applicants not using the online system, 16 of 90 (17.6%) were satisfied and 35 of 90 (38.8%) described themselves as “slightly unsatisfied” or “unsatisfied.”

Overall, when asked if applicants preferred self scheduling using the online system, 105 of the 120 (87.5%) responding stating they “agree” or “somewhat agree.” When asked if they preferred the non-online system only 13 of 121 (10.7%) stated they “agree” or “somewhat agree.” In fact, 75 of the 121 (62%) responding to this question stated they “disagreed” or “somewhat disagreed.”

In terms of travel, 84 of 119 (70.6%) applicants “agreed” or “somewhat agreed” to the statement that the online system made making their travel arrangements easier. When applicants did not use the online system, only 11 of 121 (9.1%) stated they “agreed” or “somewhat agreed” in the statement that the non-online systems made making their travel arrangements easier. Fifty-one of the 121 (42.1%) respondents stated they “disagreed” or “somewhat disagreed.”

## DISCUSSION

The results of this pilot study show that applicants prefer online systems. This result is consistent with a prior study showing high satisfaction with online interview scheduling.^1^ While we did not study this, we assume applicants prefer online scheduling because it conforms to their schedules and their access to technology. An online system can allow them to schedule or change interviews 24 hours a day. For this technologically adept cohort, an online system may be more convenient or familiar than emails and phone calls. Similarly, the online system allows applicants to rapidly change interview dates electronically to coordinate interviews at other programs. What was surprising and unexpected in our results was the time to schedule an interview with half of applicants reporting scheduling interviews within 10 minutes.

Our initial cost was $342 plus about three hours of set-up time. Based on an estimated $35/hr salary for program coordinators, our investment was $447. We estimate that prior to this system, our program coordinator spent approximately 70 hours coordinating, scheduling, and rescheduling interviews. We saved $2,450 (70hrs x $35/hr) or $5.48 for every dollar invested. We did not attempt to quantify increased satisfaction as a measure of return on investment. There may be significant variation among programs for cost savings. Some programs may have a lower cost for program coordinator salary which might lower their cost savings and return on investment.

This online system, or others similar, may substantially change the scheduling of interviews and free up program coordinator and program director time. We assumed that there was increased accuracy with the online system and there was no information ‘lost in translation’ over the phone but did not study this outcome.

The study was completed in the 2010–2011 interview season. During the 2010 interview season, the authors’ residency was one of only four EM programs using such a system based on information from the company. In addition, it was the first year the company offered the system.^3^ It is not clear how many programs are currently using online interview tools as the number of commercially available products has increased. A larger multi-center study is currently underway.

## LIMITATIONS

Of the total applicants surveyed, only 121 (or 70.8%) responded. The ones who responded may have been more pleased with the system and have been more likely to answer the survey.

Additionally, those responded knew the survey was coming from our program. The survey was conducted prior to submission of the rank lists. The unblended aspect of the survey might have biased responders into responding favorably in the hope that it might affect their position on the rank list. (The survey was anonymous and the consent stated so.)

There was no way to accurately gauge the actual time spent on task for the applicants. They did not keep a time diary. Their recall of the time spent on task may not be accurate as it may have been 1–3 weeks after they received their interview offers. We chose to not wait until after match day as the time passed since the interviews were scheduled would have been over five months.

We also acknowledge that there are frequently follow-up phone calls or emails that require program coordinator time. We feel this likely happens with any system. Our initial time savings were based on calculations for the initial scheduling and rescheduling tasks that Interview Broker automated.

Responders might inherently prefer online systems because of their facility with technology. The survey may have confounders inherent in that it was merely measuring preferences for computer-based administrative systems rather than human based systems for simple administrative tasks.

## CONCLUSION

In this pilot study, an online scheduling system was associated with higher satisfaction among applicants, including satisfaction for changing interview dates and making travel plans. We found a significant return on investment in terms of increased available administrator time.
